# Error Reduction in Leukemia Machine Learning Classification With Conformal Prediction

**DOI:** 10.1200/CCI-24-00324

**Published:** 2025-05-28

**Authors:** Mariya Lysenkova Wiklander, Dave Zachariah, Olga Krali, Jessica Nordlund

**Affiliations:** ^1^ Department of Medical Sciences, Uppsala University, Uppsala, Sweden; ^2^ SciLifeLab, Uppsala University, Uppsala, Sweden; ^3^ Department of Information Technology, Uppsala University, Uppsala, Sweden

## Abstract

**PURPOSE:**

Recent advances in machine learning have led to the development of classifiers that predict molecular subtypes of acute lymphoblastic leukemia (ALL) using RNA-sequencing (RNA-seq) data. Although these models have shown promising results, they often lack robust performance guarantees. The aim of this study was three-fold: to quantify the uncertainty of these classifiers, to provide prediction sets that control the false-negative rate (FNR), and to perform implicit error reduction by transforming incorrect predictions into uncertain predictions.

**METHODS:**

Conformal prediction (CP) is a distribution-agnostic framework for generating statistically calibrated prediction sets whose size reflects model uncertainty. In this study, we applied an extension called conformal risk control to three RNA-seq ALL subtype classifiers. Leveraging RNA-seq data from 1,227 patient samples taken at diagnosis, we developed a multiclass conformal predictor ALLCoP, which generates statistically guaranteed FNR-controlled prediction sets.

**RESULTS:**

ALLCoP was able to create prediction sets with specified FNR tolerances ranging from 7.5% to 30%. In a validation cohort, ALLCoP successfully reduced the FNR of the ALLIUM RNA-seq ALL subtype classifier from 8.95% to 3.5%. For patients whose subtype was not previously known, the use of ALLCoP was able to reduce the occurrence of empty predictions from 37% to 17%. Notably, up to 34% of the multiple-class prediction sets included the *PAX5*alt subtype, suggesting that increased prediction set size may reflect secondary aberrations and biological complexity, contributing to classifier uncertainty. Finally, ALLCoP was validated on two additional RNA-seq ALL subtype classifiers, ALLSorts and ALLCatchR.

**CONCLUSION:**

Our results highlight the potential of CP in enhancing the use of oncologic RNA-seq subtyping classifiers and also in uncovering additional molecular aberrations of potential clinical importance.

## INTRODUCTION

In the past decade, there has been an explosion in the number of diagnostic machine learning (ML) models developed for precision oncology, with the promise of delivering increasingly accurate diagnostics and personalized treatment.^1,2^ acute lymphoblastic leukemia (ALL), the most common cancer in children and a highly heterogeneous disease, has seen the development of numerous classifiers linking transcriptomic footprints to subtype-defining chromosomal aberrations.^3-7^ ALL subtypes hold prognostic value, aid in monitoring disease progression, and help determine treatment intensity.^8,9^ Before the introduction of next generation sequencing (NGS)–based diagnostics, patients with ALL were subtyped at diagnosis using techniques such as G-banding, fluorescence in situ hybridization, and reverse transcription polymerase chain reaction. At present, whole-genome sequencing and whole-transcriptome sequencing (WTS) are emerging as alternative methods to determine the subtype without a priori knowledge of the underlying genomic aberrations.^10^ However, some patients remain unclassified even with the use of WTS analysis pipelines,^11^ making ML subtyping classifiers a useful alternative. Yet, despite increasing efforts to implement data for clinical decision making in hematology,^12^ numerous challenges remain to the deployment of diagnostic ML models, ranging from insufficient regulatory frameworks for artificial intelligence (AI) to the poor clinical applicability of such models.^13,14^

**CONTEXT**

**Key Objective**
Can conformal prediction be used to reduce the error of models that use RNA-sequencing data to predict the molecular subtypes of acute lymphoblastic leukemia (ALL)?
**Knowledge Generated**
The conformal predictor ALLCoP reduced the false-negative rate of the ALLIUM classifier from 8.95% to 3.5% in a validation cohort. For patients with ALL whose subtype was not previously known, the use of ALLCoP was able to reduce the occurrence of empty predictions from 37% to 17%.
**Relevance *(F.P.-Y. Lin)***
Conformal prediction offers an alternative framework for representing machine learning outputs instead of point prediction scores. This approach has the potential to improve transparency and reduce missed diagnoses, as comprehensively demonstrated by this study, with applications extending beyond leukemia diagnostic classification.*
*Relevance section written by *JCO Clinical Cancer Informatics* Deputy Editor Frank Po-Yen Lin, PhD, FRACP, MBChB, FAIDH.

Among these challenges is the quantification of reliability and uncertainty in ML classification models. Out of the box, classifiers typically output a naïve *point prediction*, a top-scoring class or *k* top-scoring classes with no metric of uncertainty, while the underlying probabilistic scores for each class are not calibrated to empirical probability and are therefore not interpretable as confidence scores. Furthermore, traditional ML models are evaluated with population-based metrics, which do not give useful indications of uncertainty for individual patients and leave no explanation for classifier errors when they occur.^15^

Conformal prediction (CP), a distribution-agnostic and model-agnostic framework, addresses this problem by replacing point predictions with *prediction sets* containing all true classes at a user-specified probability, using an independent calibration data set to determine softmax thresholds for inclusion of classes in these sets.^16-19^ In addition to providing a mathematically proven, statistically guaranteed prediction set, CP aids in the human interpretability of ML outputs: a CP set containing a single class shows that the classifier is highly certain about the prediction, while a larger set shows a higher degree of uncertainty, and an empty set indicates that the model does not recognize the input, suggesting an out-of-distribution instance. Cresswell et al^20^ showed that the use of CP sets, with size inherently quantifying uncertainty, can improve human decision making compared with models simply outputting the *k* top-scoring classes. Finally, CP can be used for implicit error reduction: It is possible to select a lower error rate for the classifier (increased accuracy) at the expense of larger, less certain prediction sets (decreased precision).

To date, medical applications of CP remain few.^21^ A handful of studies have shown CP applied to drug discovery,^22^ disease course prediction in multiple sclerosis,^23^ lung tissue microscopy,^24^ as well as oncology, including prostate^25^ and breast cancer^26^ classification. A 2006 study applied CP to a support vector machine trained to predict five ALL and three AML subtypes using microarray-generated gene expression (GEX) data up to a 95% confidence level.^27^ However, to our knowledge, to date, there have been no applications of CP to RNA-sequencing (RNA-seq) classifiers for leukemia or any other cancer.

In this study, we applied CP to three ALL subtype classifiers using RNA-seq data from 1,227 patients from five different ALL cohorts^3,28-30^ encompassing 14 ALL subtypes. A conformal predictor, ALLCoP, was first calibrated and cross-validated using predictions from the ALLIUM classifier.^3^ In a validation data set, ALLCoP was able to reduce the false-negative rate (FNR) from 8.95% to 3.5%. ALLCoP was then used to create prediction sets for 126 samples whose subtype was unknown at diagnosis. Finally, ALLCoP was validated on two additional classifiers, ALLSorts^4^ and ALLCatchR.^5^

## METHODS

### Data

Publicly available GEX counts were obtained from five different studies^3,28-30^ comprising RNA-seq data from a total of 1,227 diagnostic pediatric and young adult ALL samples. After preprocessing, 1,042 samples were subjected to ALL subtype prediction using ALLIUM,^3^ while 292 samples were subjected to the ALLCatchR^5^ and ALLSorts^4^ models. Of the ALLIUM predictions, samples whose subtype was denoted as unknown in the original studies (n = 126) and samples with multiple known subtypes (n = 65) were analyzed separately, leaving 851 samples for ALLCoP calibration and validation. The model and data preprocessing are specified in the Data Supplement, together with cohort details (Table S1); batch effects and correction are illustrated in the Data Supplement (Figs S1 and S2).

### Conformal Prediction

The conformal predictor ALLCoP used the classifier outputs as inputs, both for calibration and the subsequent formation of prediction sets (Data Supplement, Fig S3). We used *split CP*, which requires calibration and validation using a data set independent from the training data set of the underlying model,^19^ and *conformal risk control*, which allows the application of CP to data sets with multiple true classes.^31^ The latter produces prediction sets at a user-defined tolerance on the FNR, denoted α, and an FNR-controlling softmax threshold value*, lamhat*, above which classes are included in the prediction sets (Data Supplement, Fig S4).

We first validated ALLCoP. Using the ALLIUM predictions for all samples with a single known subtype (n = 851), a series of cross-validation experiments were configured whereby each experiment consisted of multiple runs and in each run, the prediction data set was shuffled and split: 90% of predictions were used for calibration of the conformal predictor using the conformal risk control^31^ algorithm and 10% were used for validation. ALLCoP was then recalibrated in different configurations to generate prediction sets for subsets of the data (Data Supplement).

## RESULTS

### Uncertainty in ALL Subtype Classification

**FIG 1.**
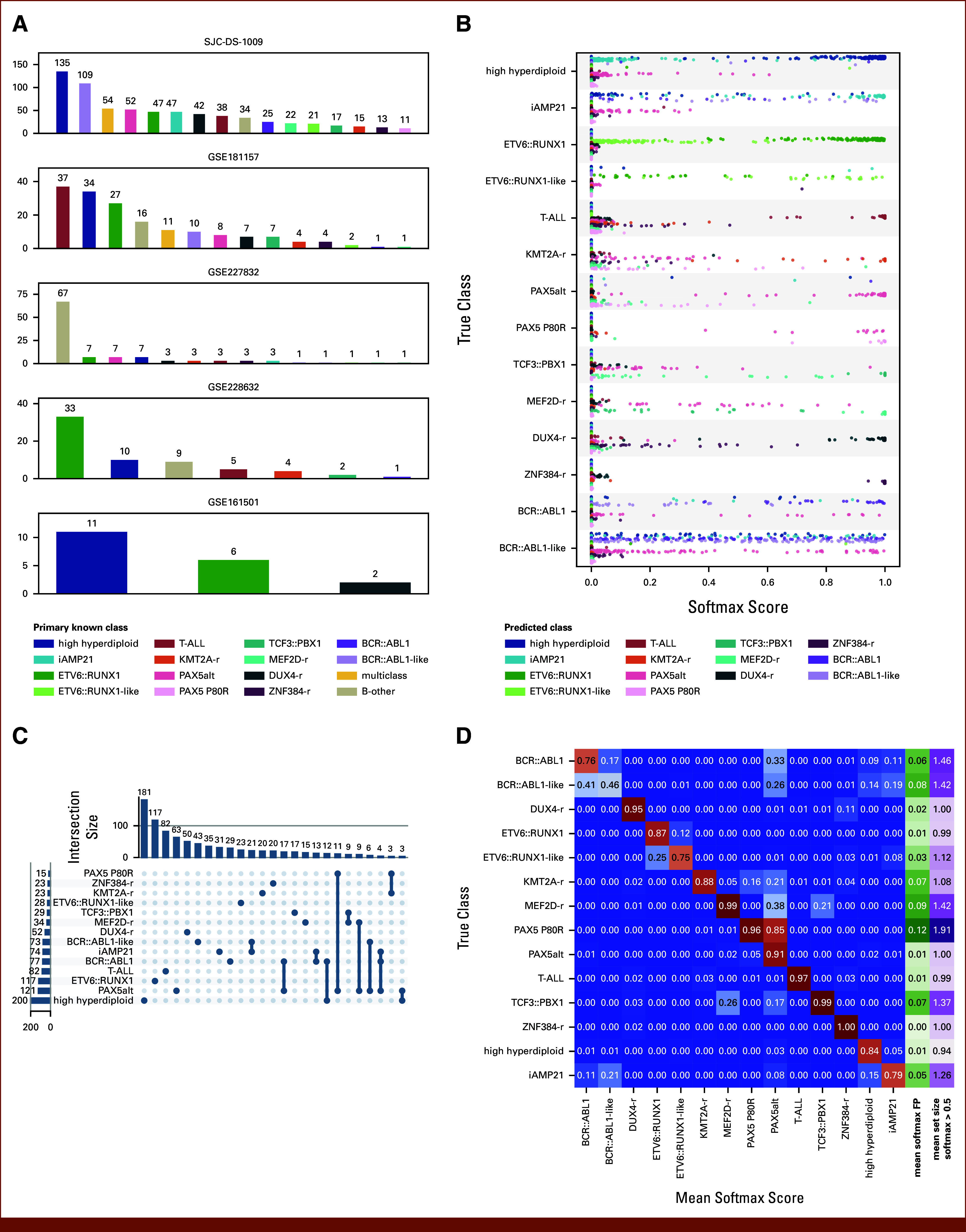
RNA-sequencing data sets and ALLIUM predictions used as input for ALLCoP. (A) The subtype distributions for the 1,042 samples used for the ALLIUM implementation of ALLCoP. The remaining panels visualize ALLIUM predictions for the 851 samples with a single known subtype, including (B) softmax scores output by the model, stratified by true subtype and colored by predicted subtype; (C) the class membership of ALLIUM prediction sets, formed using a softmax threshold of 0.5; and (D) a heatmap mapping each true subtype to the mean softmax score per predicted subtype, with the green column showing the mean softmax scores of false positives and the purple column showing the mean size of the ALLIUM prediction sets with softmax threshold = 0.5.

Our aim was to investigate whether applying CP could quantify the uncertainty and reduce the error rate of RNA-seq ALL subtype classifiers. We applied ALLIUM^3^ to 1,042 orthogonal RNA-seq samples from patients with ALL from five studies^3,28-30^ (Data Supplement, Table S2). The distribution of their known molecular subtypes is shown in Figure 1A.

For the 851 samples with a single known subtype, the softmax scores of the ALLIUM predictions were unequally distributed by true subtype, with, for example, T-ALL and *ZNF384*-r showing consistently high scores for the correct subtypes only and with other subtypes such as *BCR::ABL1* and *BCR::ABL1-like* receiving high scores in more than 1 class (Fig 1B).

Using a threshold of 0.5, we formed prediction sets comprised of all subtypes whose ALLIUM softmax score surpassed this value. Of the samples, 691 (81.2%) resulted in single-class prediction sets, most frequently high hyperdiploid (n = 181) or *ETV6::RUNX1* (n = 117). However, some subtypes were consistently found to co-occur with others, such as PAX5 P80R; 78.6% of prediction sets containing this class also contained the subtype *PAX5*alt (Fig 1C).

ALLIUM has difficulty distinguishing between these two subtypes, although the true subtype typically scored higher, with true PAX5 P80R receiving a mean softmax score of 0.96 versus 0.85 for *PAX5*alt. Again, using a softmax score cutoff of 0.5, the mean prediction set size, meaning the number of classes predicted per individual sample, varied per subtype, with the highest mean set sizes observed for PAX5 P80R, *BCR::ABL1*, *BCR::ABL1*-like, and *MEF2D*-r (Fig 1D).

### Empirical Error Rate Selection and FNR Validation

Next, we developed and applied a conformal predictor (ALLCoP) to the ALLIUM predictions (softmax scores), to (1) evaluate error reduction to predefined levels and (2) enable reporting of multiple potentially true subtype calls.^32^

**FIG 2.**
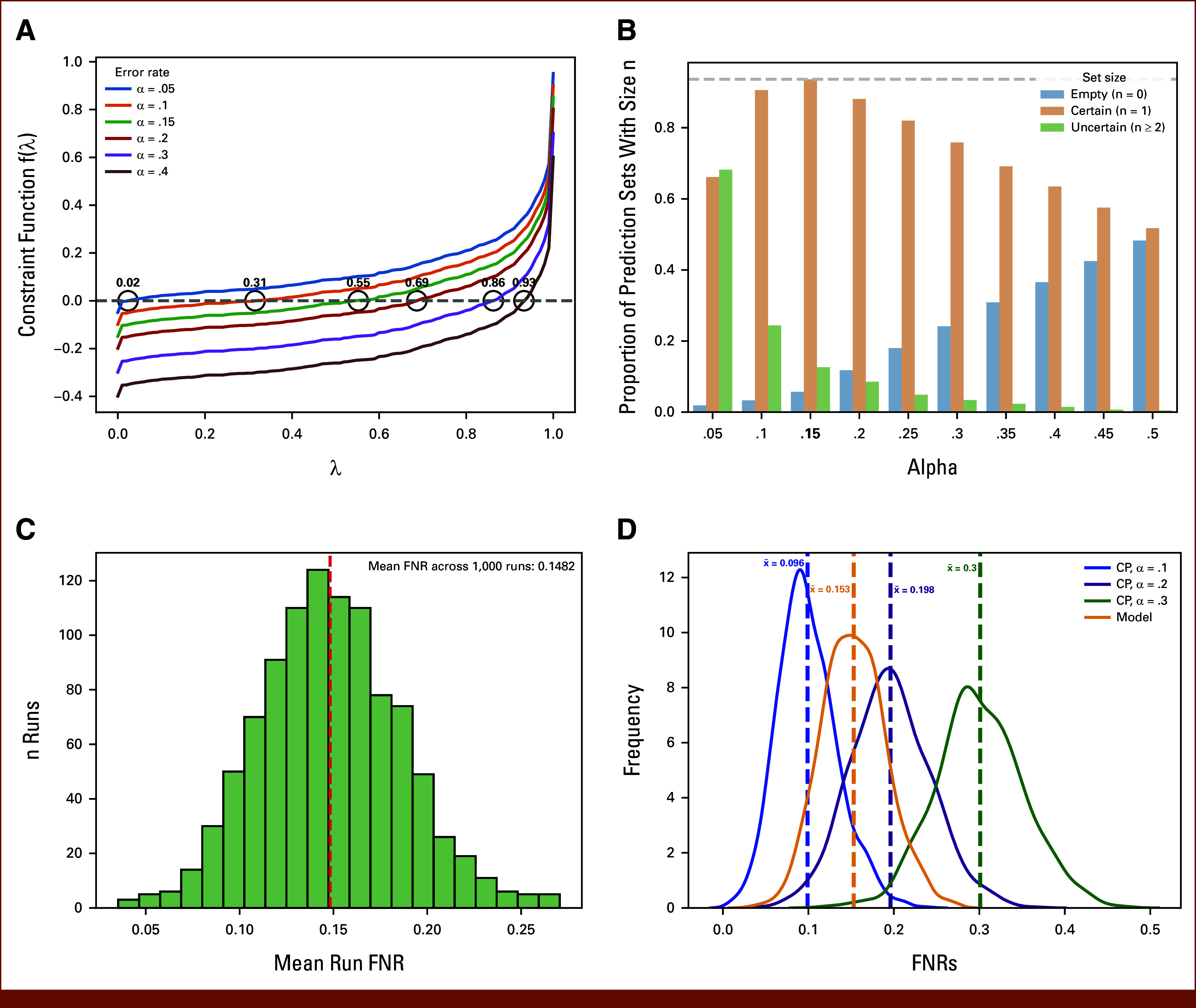
Empirical ALLCoP error rate selection and FNR validation for the ALLIUM classifier. (A) The output of the constraint function that selects the FNR-controlling model softmax threshold *lamhat* for a range of error rate values α. (B) From an experiment of 1000 ALLCoP runs for a range of α values, shown are the proportions of resulting prediction sets that were empty, certain (size = 1), or uncertain (size ≥2). The gray dashed line and bolded number indicate the α value at which the highest proportion of certain prediction sets occurs. (C) The mean FNRs of prediction sets produced in an experiment of 1000 ALLCoP runs with error rate α = .15, empirically testing the overall FNR. (D) The FNRs of the ALLCoP prediction sets produced at a range of α values, versus the FNR of the uncalibrated ALLIUM classifier outputs, defined as a set containing the single top-scoring subtype, in yellow. FNR, false-negative rate.

Using ALLCoP, we created prediction sets using a range of FNR tolerance α levels (0.05-0.50) and corresponding FNR-controlling softmax threshold values, lamhat, above which classes are included in the output prediction sets (Fig 2A; Data Supplement, Table S3).

At α = .15, ALLCoP produced the highest proportion of high certainty, single-class prediction sets (81.6%), and relatively few sets representing no prediction (5.7% empty sets) or uncertain prediction (12.7% of sets with size ≥2; Fig 2B). The mean FNR of the prediction sets was 14.82%, in line with the selected value of α = .15 (Fig 2C).

In experiments of 1,000 runs per α value, the FNR of ALLCoP prediction sets with α∈{0.1,0.2,0.3} were then compared against the FNR of the uncalibrated ALLIUM classifier outputs. In line with the conformal statistical guarantee, the FNRs of the ALLCoP prediction sets were 9.6%, 19.8%, and 30.0%, while the mean FNR for ALLIUM was 15.3% (Fig 2D). FNRs for ALLCoP at all three error rates were at or below the corresponding α values, indicating that the ALLCoP statistical guarantee holds across a diverse range of conditions, irrespective of the underlying model's performance.

### Performance by ALL Subtype

Next, we evaluated ALLCoP performance by true ALL subtype. In order to obtain generalizations across the entire dataset, we cross-validated with 10 thousand runs per α value with α∈{0.075,0.1,0.15} using predictions from the 851 single-subtype samples. We evaluated the FNR and set sizes of the resulting prediction sets, stratified by subtype.

**FIG 3.**
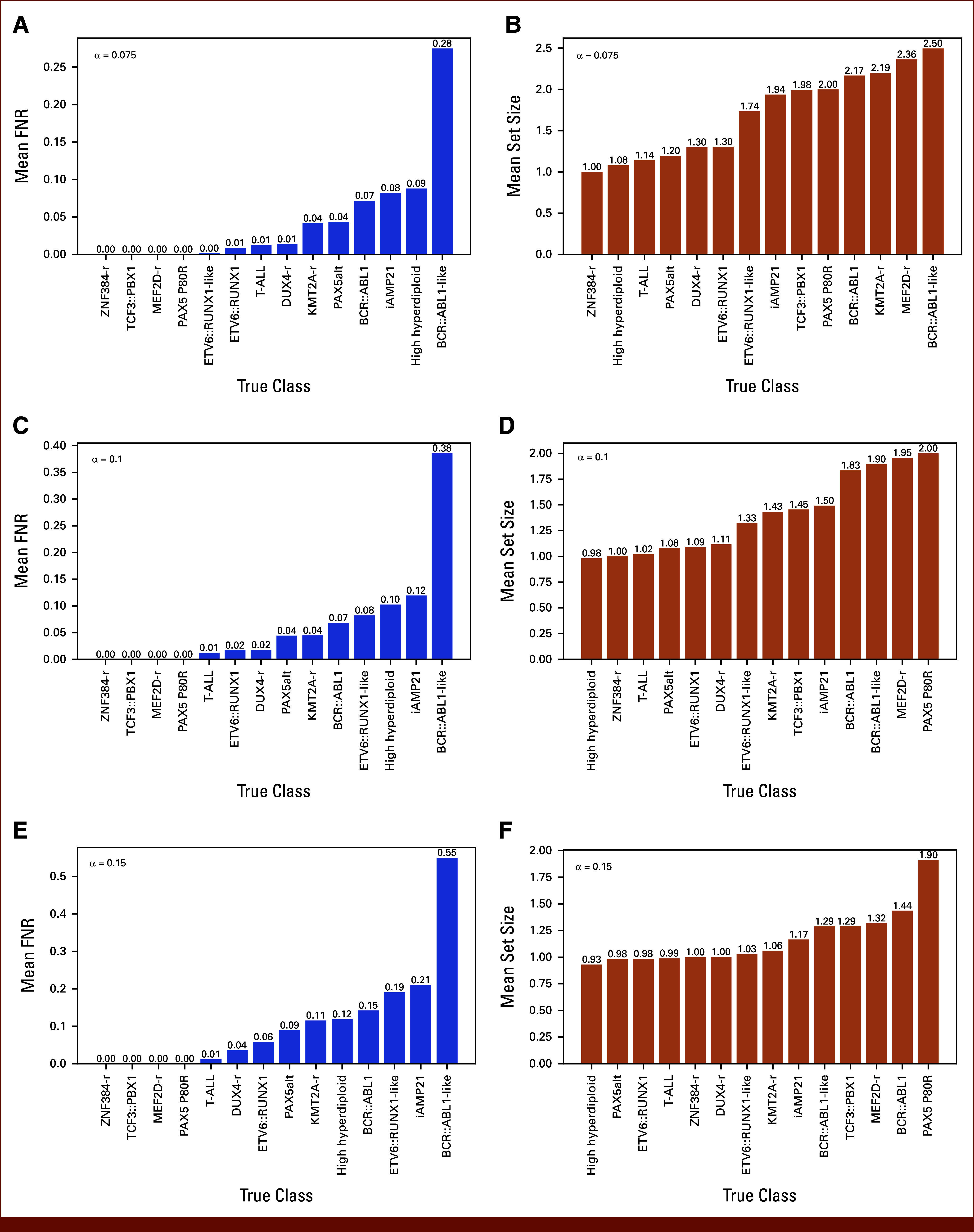
ALLCoP prediction sets generated from ALLIUM predictions, by subtype. (A) Mean FNRs for prediction sets, α = .075. (B) Mean sizes for prediction sets, α = .075. (C) Mean FNRs for prediction sets, α = .1. (D) Mean sizes for prediction sets, α = .1. (E) Mean FNRs for prediction sets, α = .15. (F) Mean sizes for prediction sets, α = .15. FNR, false-negative rate.

At α = .075, the FNRs for the different subtypes ranged from 0% to 28% (Fig 3A), while the set sizes ranged from 1.0 to 2.50 (Fig 3B). At α = .1, the FNRs ranged from 0% to 38% (Fig 3C) and set sizes from 0.98 to 2.0 (Fig 3D). At α = .15, the FNRs ranged from 0% to 55% (Fig 3E) and set sizes from 0.93 to 1.90 (Fig 3F).

For subtypes *ZNF384*-r, *TCF3::PBX1*, and *MEF2D*-r, the FNR remained 0% across all three α values, indicating high classifier certainty, with the correct class always included in the respective prediction sets. *BCR::ABL1*-like had the highest FNR across all α values, from 28% at α = .075 to 55% at α = .15, and the highest set size at α = .075 (2.50), indicating that high-uncertainty classes are increasingly included in prediction sets at lower α values, at the cost of larger prediction sets.

### Implicit Error Reduction in a Validation Data Set

After cross-validating the conformal guarantee in ALLCoP and assessing its performance across ALL subtypes, we then recalibrated instances of ALLCoP to produce prediction sets for three data subsets: first, for samples with a single known subtype; second, for samples with multiple known subtypes; and finally, for samples of unknown subtype. We aimed to show that CP can be used to reduce error in validation data with a known subtype and to produce fewer empty predictions for samples with an unknown subtype.

Using ALLIUM predictions from the St Jude Cloud samples with a single known subtype (n = 594),^28^ we recalibrated ALLCoP with α∈{0.075,0.1,0.15} and obtained prediction sets for the samples with a single known subtype from the other cohorts (n = 257)^3,29,30^ (Data Supplement, Table S4).

**FIG 4.**
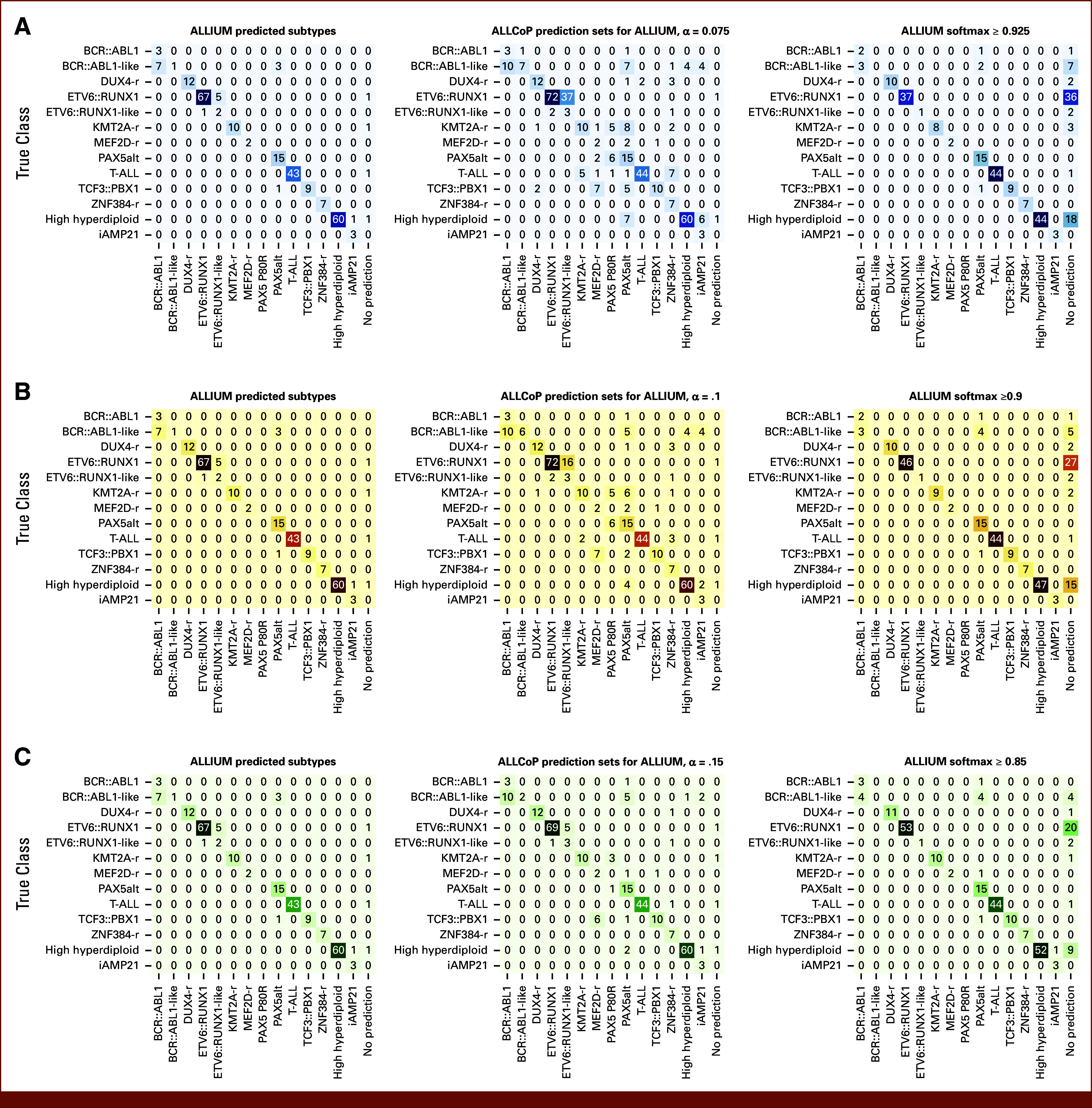
Concordance between ALLIUM single-class point predictions, ALLCoP sets, and sets of classes where the softmax score was >1-α in the validation data set with a single known subtype, at preselected FNRs of (A) α = .075, (B) α = .1, and (C) α = .15. FNR, false-negative rate.

In this validation set, 234 ALLIUM predictions were correct, 19 were wrong, and four were empty. Of these 23 samples with incorrect or empty predictions, ALLCoP prediction sets contained the correct class for six patients at α = .15, 13 patients at α = .1, and 14 patients at α = .075. In this validation set, the uncalibrated ALLIUM model had an FNR of 8.95%, which was reduced to 6.61% at α = .15, 3.89% at α = .10, and 3.50% at α = .075; the trade-off was an increasing mean set size: 1.11 at α = .15, 1.31 at α = .10, and 1.53 at α = .075 (Data Supplement, Table S5). A comparison between ALLIUM class predictions, ALLCoP sets, and sets containing classes where the softmax score was >1-α is shown for α = .075 (Fig 4A), α = .1 (Fig 4B), and α = .15 (Fig 4C). The mean FNR and mean set size are stratified by subtype in the Data Supplement (Table S6). Notably, the FNR of *BCR::ABL1*-like, which was 90.9% in the uncalibrated ALLIUM output, was reduced to 36.36% at α = .075. Also of note, *PAX5*alt frequently co-occurred with other subtypes, appearing in 10 of 29 multiclass prediction sets at α = .15 (34.48%), 20 of 65 at α = .10 (30.77%), and 31 of 103 (30.10%) at α = .075.

We then used the same conformal predictors to obtain prediction sets for samples with multiple known subtypes (n = 65)^28,30^ (Data Supplement, Table S7). Briefly, the uncalibrated ALLIUM model had an FNR of 61.54%, which was reduced to 23.85% at α = .075 (Data Supplement, Table S8; performance by subtype in the Data Supplement, Table S9). The prediction sets are visualized in the Data Supplement (Fig S5).

### Prediction Sets for Unknown Subtype Patients

We calibrated three instances of ALLCoP using all patients with a single known subtype (n = 851) at error rates α∈{0.075,0.1,0.15} and used them to generate prediction sets for samples with an unknown subtype from all studies (n = 126; Data Supplement, Table S10).

**FIG 5.**
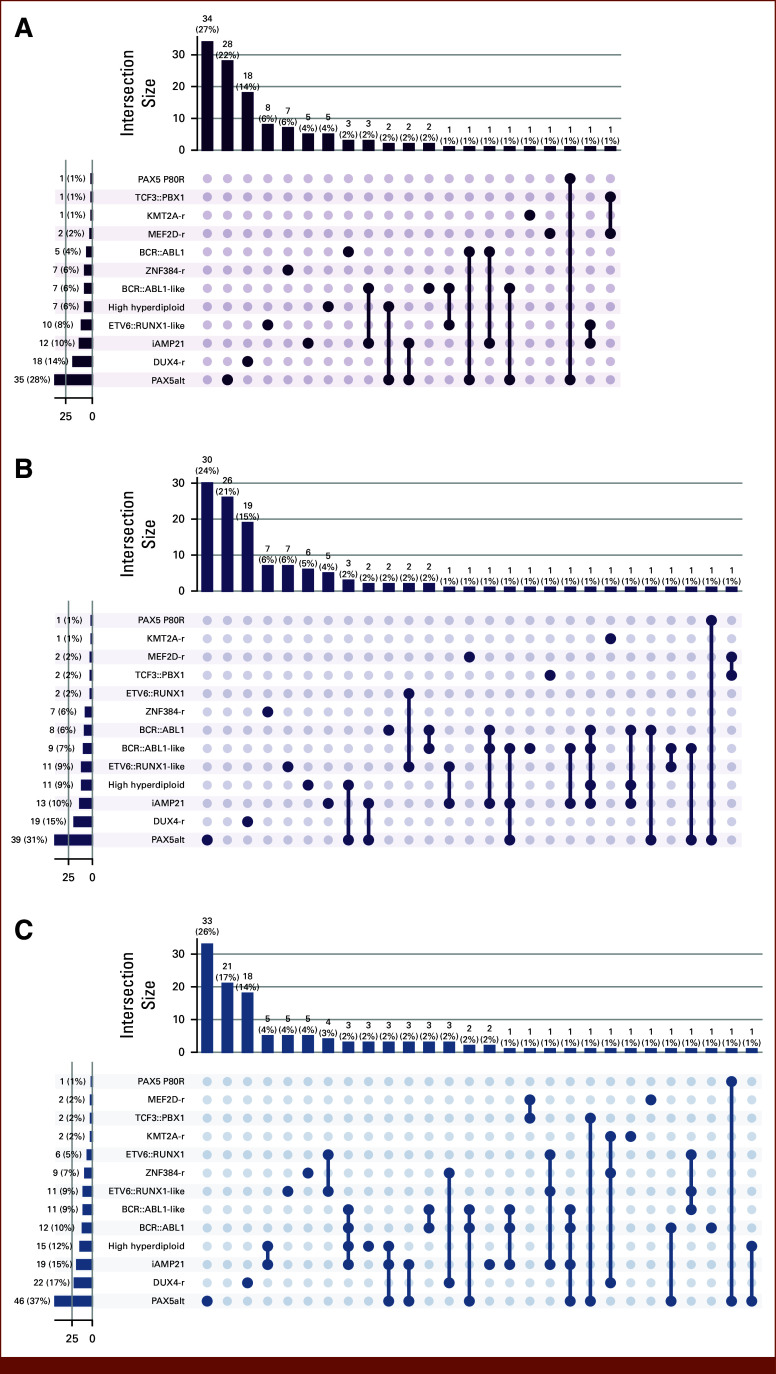
ALLCoP prediction sets for 126 patients with previously unclassified B-ALL using predictions from ALLIUM. The upset plots represent class membership counts, with unattached dots representing single-class prediction sets and connected dots representing multiclass sets. Visualized are prediction sets for preselected FNRs of (A) α = .15, resulting in 34 empty sets, 78 certain sets, and 14 uncertain sets; (B) α = .10, resulting in 26 empty sets, 80 certain sets, and 20 uncertain sets; and (C) α = .075, resulting in 21 empty sets, 69 certain sets, and 36 uncertain sets. FNR, false-negative rate.

Using the uncalibrated model, ALLIUM issued predictions for 97 patients at the default model softmax threshold of 0.5, leaving 29 empty predictions (23%). At α = .15, 34 of the ALLCoP prediction sets were empty (27%; Fig 5A), but at α = .10, this number dropped to 26 empty sets (21%; Fig 5B), and at α = .075, only 21 of the sets were empty (17%; Fig 5C). Across all three error rates, the most commonly predicted subtypes were *PAX5*alt, *DUX4*-r, and iAMP21.

### Validation on Additional RNA-Seq ALL Subtype Classifiers

**FIG 6.**
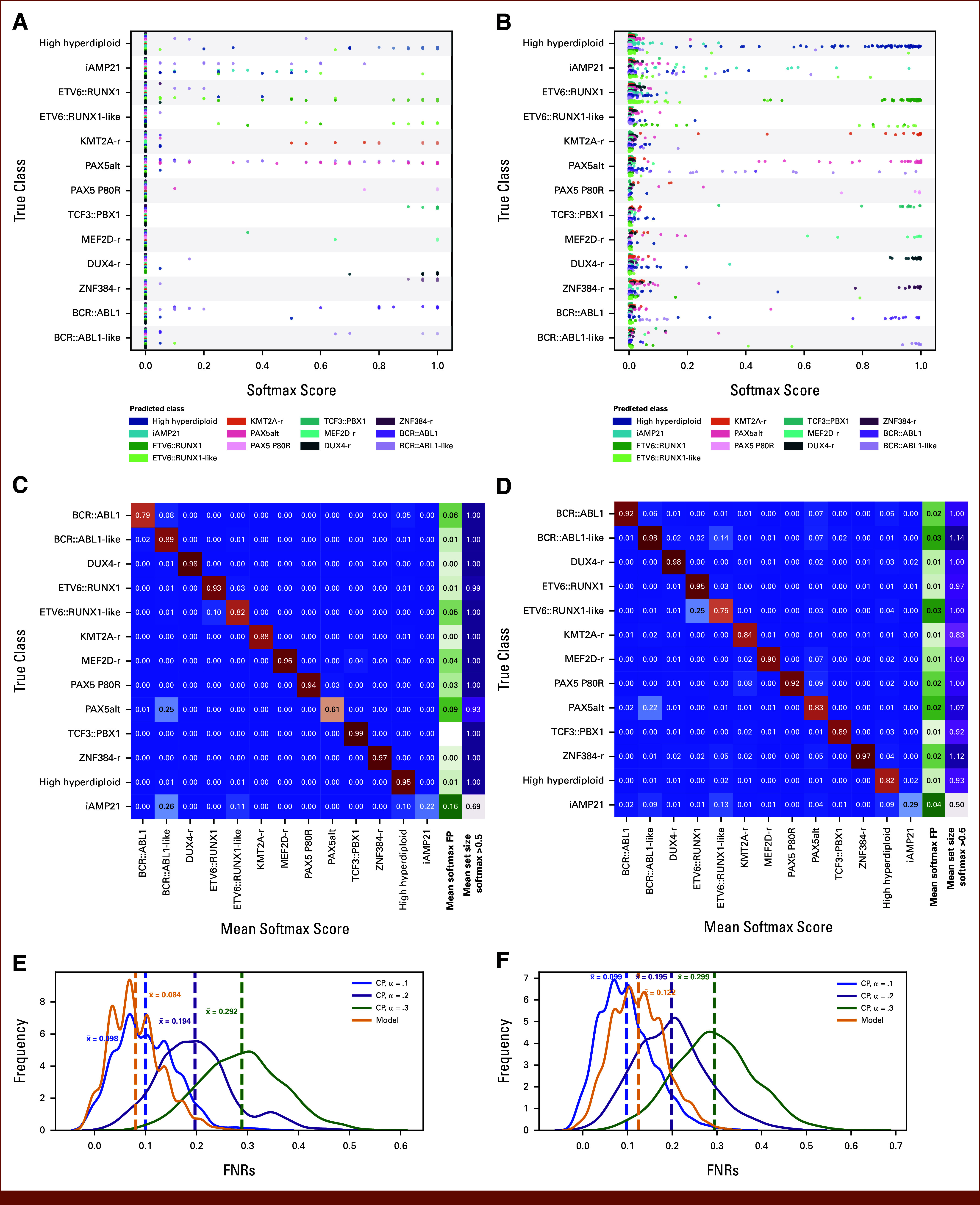
Softmax predictions and ALLCoP results for two ALL RNA-seq classifiers, ALLCatchR and ALLSorts. All softmax scores output by the model, stratified by true subtype and colored by predicted subtype, for (A) ALLCatchR and (B) ALLSorts. Heatmaps mapping each true subtype to the mean softmax score per predicted subtype, with the green column showing the mean softmax scores of false positives and the purple column showing the mean size of the prediction sets with softmax threshold = 0.5, for (C) ALLCatchR and (D) ALLSorts. FNR of ALLCoP prediction sets versus uncalibrated model outputs for (E) ALLCatchR and (F) ALLSorts, with the uncalibrated model outputs in yellow. FNR, false-negative rate; RNA-seq, RNA-sequencing.

ALLIUM is one of numerous RNA-seq classifiers for molecular subtype determination in ALL.^4-6^ To evaluate the generalizability of ALLCoP to other classifiers, we selected two other models, ALLCatchR^5^ and ALLSorts,^4^ and generated predictions for samples that were not used for training these models^3,29^ (Data Supplement, Fig S6; Data Supplement, Tables S11-S12). The softmax scores generated by ALLCatchR resulted in discrete distributions (Fig 6A). The subtypes exhibiting the highest uncertainty were iAMP21 (high softmax scores for *BCR::ABL1*-like, *ETV6::RUNX1*-like, and high hyperdiploid) and *PAX5*alt (high softmax scores for *BCR::ABL1*-like). Similar to ALLCatchR, ALLSorts was most uncertain in predicting iAMP21, often confusing it for the same classes as ALLCatchR (Fig 6B). ALLCatchR generated largely certain predictions, with the mean prediction set size (using a softmax threshold of 0.5) never surpassing 1.0 and remaining over 0.99 for all classes, except *PAX5*alt (0.93) and iAMP21 (0.69; Fig 6C). Similarly, ALLSorts had a mean set size of over 0.90 for all classes, except *KMT2A*-r (0.83) and iAMP21 (0.50), although it had more uncertain sets, with a mean set size >1.0 for *BCR::ABL1*-like (1.14), *ZNR384*-r (1.12), and *PAX5*alt (1.07; Fig 6D). In ALLSorts, as with ALLIUM prediction sets, *PAX5*alt was the subtype most frequently observed in multiclass predictions, although in the discrete model outputs of ALLCatchR, this was not observed.

Next, we applied ALLCoP to the softmax scores and evaluated the output (Data Supplement, Tables S13-S14). Similarly to ALLIUM, ALLCoP produced prediction sets at defined FNRs for AllCatchR and ALLSorts (Figs 6E and 6F). Empirical error rate selection was performed for the two classifiers, showing an optimal error rate of α = .1 for ALLCatchR (softmax threshold of 0.44) and α = .05 for ALLSorts (softmax threshold of 0.18; Data Supplement, Fig S7). These results further demonstrate the robustness and generalizability of ALLCoP for RNA-seq ALL classifiers.

## DISCUSSION

In this study, we present the first application of conformal risk control, to our knowledge, to RNA-seq–based ML classification of ALL. Recent and forthcoming legislation, such as the EU AI Act,^33^ emphasizes regulatory requirements including transparency, explainability, and accountability in AI systems.^34,35^ In this context, the quantification of model robustness and confidence is essential.^36^ This necessitates methodologies beyond the uncalibrated softmax outputs typically generated by classifiers, which do not reflect empirical probabilities.^37,38^

CP, by providing statistical performance certification for predictions, supports future clinical translation of RNA-seq classifiers. At present, a small subset of International Consensus Classification (ICC)–recognized subtypes^8,39^ are used to guide therapeutic decision making for ALL treatment. However, recent studies have demonstrated the utility of increasingly fine-grained molecular subtyping both for predicting outcomes and tailoring treatment intensity.^40^ This research landscape is fluid, with both the continuous emergence of novel subtypes^41^ and the refinement of existing ICC subtypes.^42^ Established subtypes that show clinical significance include *PAX5*alt, *BCR::ABL1*-like, *ETV6::RUNX1*-like, and *MEF2D*-r,^43^ which are among the classes whose ALLIUM predictions exhibited high levels of uncertainty in this study. CP can identify ambiguous cases among these clinically relevant subtypes, flagging them for further human assessment and contributing to more reliable classification outcomes. As such, this framework has the potential to facilitate the translation of refined molecular risk stratification to clinical use and to eventually improve patient outcomes.

The variability of CP set size, referred to as *set adaptivity*, serves as a valuable metric of uncertainty. This reflects both the performance of the classifier and the irreducible uncertainty arising from the inherent complexity of the features being classified. In our study, the *PAX5*alt subtype most frequently appeared in multiclass prediction sets generated using ALLIUM^3^ and ALLSorts.^4^ Given that alterations in the *PAX5* gene are observed in over one third of patients with B-cell precursor ALL, as reported in prior studies,^32,44^ the multiclass CP sets containing *PAX5*alt may not solely indicate classifier uncertainty but could also point to biologically relevant secondary aberrations with potential clinical importance.

Finally, we explored the concept of implicit error reduction within the framework of ALL RNA-seq classifiers. In the validation cohorts, we found that lowering the α value resulted in the expected reduction in the mean FNR of the prediction sets. Likewise, for samples with unknown subtypes, a lower α value resulted in fewer empty prediction sets, demonstrating improved model utility.

However, this approach involves a trade-off: Reducing the α value increases the likelihood of including the true class in the prediction sets but results in larger and less precise sets.^45^ The ability to select an α value tailored to specific requirements offers significant flexibility. For instance, a higher tolerance for error may be acceptable in research contexts where exploratory analysis is prioritized. Conversely, high-stakes applications demand stricter error rates to ensure reliable and actionable predictions.^46,47^ Obtaining regulatory approval for medical advice applications, for instance, may require performing risk analysis to determine an appropriate error rate,^48^ although AI best practices and regulatory requirements are still in development.^49^ The adaptability of CP underscores its potential as a versatile tool for integrating ML into both research and clinical workflows, balancing precision with reliability on the basis of user-defined thresholds.

In summary, this study demonstrates the potential of CP to enhance the robustness, transparency, and adaptability of RNA-seq–based ML classifiers for ALL subtyping. By addressing both predictive uncertainty and error management, our findings pave the way for integrating advanced AI methodologies into clinical workflows while aligning with emerging regulatory requirements.

## Data Availability

A data sharing statement provided by the authors is available with this article at DOI https://doi.org/10.1200/CCI-24-00324. All data^3,28-30^ (Data Supplement, Table S1) and code^50-53^ (Data Supplement, Table S15) used in this study are publicly available.

## PREPRINT VERSION

Preprint available on https://www.biorxiv.org/content/10.1101/2024.12.11.627902v1.

## References

[1] Bhinder B, Gilvary C, Madhukar NS, et al: Artificial intelligence in cancer research and precision medicine. Cancer Discov 11:900-915, 2021 33811123 10.1158/2159-8290.CD-21-0090 PMC8034385

[2] Swanson K, Wu E, Zhang A, et al: From patterns to patients: Advances in clinical machine learning for cancer diagnosis, prognosis, and treatment. Cell 186:1772-1791, 2023 36905928 10.1016/j.cell.2023.01.035

[3] Krali O, Marincevic-Zuniga Y, Arvidsson G, et al: Multimodal classification of molecular subtypes in pediatric acute lymphoblastic leukemia. Npj Precis Oncol 7:131, 2023 38066241 10.1038/s41698-023-00479-5 PMC10709574

[4] Schmidt B, Brown LM, Ryland GL, et al: ALLSorts: An RNA-seq subtype classifier for B-cell acute lymphoblastic leukemia. Blood Adv 6:4093-4097, 2022 35482550 10.1182/bloodadvances.2021005894 PMC9327546

[5] Beder T, Hansen B-T, Hartmann AM, et al: The gene expression classifier ALLCatchR identifies B-cell precursor ALL subtypes and underlying developmental trajectories across age. HemaSphere 7:e939, 2023 37645423 10.1097/HS9.0000000000000939 PMC10461941

[6] Hu Z, Jia Z, Liu J, et al: MD-ALL: An integrative platform for molecular diagnosis of B-acute lymphoblastic leukemia. Haematologica 109:1741-1754, 2024 37981856 10.3324/haematol.2023.283706 PMC11141650

[7] Gu A, Schmidt B, Lonsdale A, et al: TALLSorts: A T-cell acute lymphoblastic leukemia subtype classifier using RNA-seq expression data. Blood Adv 7:7402-7406, 2023 37903323 10.1182/bloodadvances.2023010385 PMC10758738

[8] Duffield AS, Mullighan CG, Borowitz MJ: International Consensus Classification of acute lymphoblastic leukemia/lymphoma. Virchows Arch 482:11-26, 2023 36422706 10.1007/s00428-022-03448-8 PMC10646822

[9] Iacobucci I, Mullighan CG: Genetic basis of acute lymphoblastic leukemia. J Clin Oncol 35:975-983, 2017 28297628 10.1200/JCO.2016.70.7836 PMC5455679

[10] Wadensten E, Wessman S, Abel F, et al: Diagnostic yield from a nationwide implementation of precision medicine for all children with cancer. JCO Precis Oncol 10.1200/PO.23.00039 PMC10581599 37384868

[11] Hu Z, Kovach AE, Yellapantula V, et al: Transcriptome sequencing allows comprehensive genomic characterization of pediatric B-acute lymphoblastic leukemia in an academic clinical laboratory. J Mol Diagn 26:49-60, 2024 37981088 10.1016/j.jmoldx.2023.09.013 PMC10773144

[12] Edsjö A, Lindstrand A, Gisselsson D, et al: Building a precision medicine infrastructure at a national level: The Swedish experience. Camb Prisms Precis Med 1:e15, 2023 10.1017/pcm.2023.3 PMC10953755 38550923

[13] Kelly CJ, Karthikesalingam A, Suleyman M, et al: Key challenges for delivering clinical impact with artificial intelligence. BMC Med 17:195, 2019 31665002 10.1186/s12916-019-1426-2 PMC6821018

[14] AI diagnostics need attention. Nature 555:285, 2018 10.1038/d41586-018-03067-x 29542717

[15] Banerji CRS, Chakraborti T, Harbron C, et al: Clinical AI tools must convey predictive uncertainty for each individual patient. Nat Med 29:2996-2998, 2023 37821686 10.1038/s41591-023-02562-7

[16] Gammerman A, Vovk V, Vapnik V: Learning by transduction, in Cooper GF (ed): Uncertainty in artificial intelligence: proceedings of the fourteenth conference (1998), July 24-26, 1998, University of Wisconsin, Madison, WI. San Francisco, CA, Morgan Kaufmann, 1998

[17] Vovk V, Gammerman A, Shafer G: Algorithmic Learning in a Random World. New York, NY, Springer-Verlag, 2005. http://link.springer.com/10.1007/b106715

[18] Shafer G, Vovk V: A tutorial on conformal prediction, 2007. https://arxiv.org/abs/0706.3188

[19] Angelopoulos AN, Bates S: A gentle introduction to conformal prediction and distribution-free uncertainty quantification. 2021. https://arxiv.org/abs/2107.07511

[20] Cresswell JC, Sui Y, Kumar B, et al: Conformal prediction sets improve human decision making, 2024. https://arxiv.org/abs/2401.13744

[21] Vazquez J, Facelli JC: Conformal prediction in clinical medical sciences. J Healthc Inform Res 6:241-252, 2022 35898853 10.1007/s41666-021-00113-8 PMC9309105

[22] Alvarsson J, Arvidsson McShane S, Norinder U, et al: Predicting with confidence: Using conformal prediction in drug discovery. J Pharm Sci 110:42-49, 2021 33075380 10.1016/j.xphs.2020.09.055

[23] Sreenivasan AP, Vaivade A, Noui Y, et al: Conformal prediction enables disease course prediction and allows individualized diagnostic uncertainty in multiple sclerosis. npj Digit Med 8:224, 2025 40275055 10.1038/s41746-025-01616-z PMC12022056

[24] Wieslander H, Harrison PJ, Skogberg G, et al: Deep learning with conformal prediction for hierarchical analysis of large-scale whole-slide tissue images. IEEE J Biomed Health Inform 25:371-380, 2021 32750907 10.1109/JBHI.2020.2996300

[25] Olsson H, Kartasalo K, Mulliqi N, et al: Estimating diagnostic uncertainty in artificial intelligence assisted pathology using conformal prediction. Nat Commun 13:7761, 2022 36522311 10.1038/s41467-022-34945-8 PMC9755280

[26] Lambrou A, Papadopoulos H, Gammerman A: Evolutionary conformal prediction for breast cancer diagnosis, in 2009 9th International Conference on Information Technology and Applications in Biomedicine. Larnaka, Cyprus, IEEE, 2009, pp 1-4. http://ieeexplore.ieee.org/document/5394447/

[27] Bellotti T, Luo Z, Gammerman A: Reliable classification of childhood acute leukaemia from gene expression data using confidence machines, in 2006 IEEE International Conference on Granular Computing. Atlanta, GA, IEEE, 2006, pp 148-153. http://ieeexplore.ieee.org/document/1635774/

[28] McLeod C, Gout AM, Zhou X, et al: St. Jude Cloud: A pediatric cancer genomic data-sharing ecosystem. Cancer Discov 11:1082-1099, 2021 33408242 10.1158/2159-8290.CD-20-1230 PMC8102307

[29] Diedrich JD, Dong Q, Ferguson DC, et al: Profiling chromatin accessibility in pediatric acute lymphoblastic leukemia identifies subtype-specific chromatin landscapes and gene regulatory networks. Leukemia 35:3078-3091, 2021 33714976 10.1038/s41375-021-01209-1 PMC8435544

[30] Tran TH, Langlois S, Meloche C, et al: Whole-transcriptome analysis in acute lymphoblastic leukemia: A report from the DFCI ALL Consortium Protocol 16-001. Blood Adv 6:1329-1341, 2022 34933343 10.1182/bloodadvances.2021005634 PMC8864659

[31] Angelopoulos AN, Bates S, Fisch A, et al: Conformal Risk Control, 2022. https://arxiv.org/abs/2208.02814

[32] Familiades J, Bousquet M, Lafage-Pochitaloff M, et al: PAX5 mutations occur frequently in adult B-cell progenitor acute lymphoblastic leukemia and PAX5 haploinsufficiency is associated with BCR-ABL1 and TCF3-PBX1 fusion genes: A GRAALL study. Leukemia 23:1989-1998, 2009 19587702 10.1038/leu.2009.135

[33] Artificial Intelligence Act. 2024. http://data.europa.eu/eli/reg/2024/1689/oj

[34] Gyevnar B, Ferguson N, Schafer B: Bridging the Transparency Gap: What Can Explainable AI Learn From the AI Act? 2023. https://arxiv.org/abs/2302.10766

[35] He Z, Zhang R, Diallo G, et al: Editorial: Explainable artificial intelligence for critical healthcare applications. Front Artif Intell 6:1282800, 2023 37771610 10.3389/frai.2023.1282800 PMC10523392

[36] Sousa S, Paredes S, Rocha T, et al: Machine learning models’ assessment: Trust and performance. Med Biol Eng Comput 62:3397-3410, 2024 38849699 10.1007/s11517-024-03145-5 PMC11485107

[37] Filho TS, Song H, Perello-Nieto M, et al: Classifier calibration: A survey on how to assess and improve predicted class probabilities. 2021. https://arxiv.org/abs/2112.10327

[38] Guo C, Pleiss G, Sun Y, et al: On Calibration of Modern Neural Networks, 2017. https://arxiv.org/abs/1706.04599

[39] Toft N, Birgens H, Abrahamsson J, et al: Results of NOPHO ALL2008 treatment for patients aged 1–45 years with acute lymphoblastic leukemia. Leukemia 32:606-615, 2018 28819280 10.1038/leu.2017.265

[40] Chang T-C, Chen W, Qu C, et al: Genomic determinants of outcome in acute lymphoblastic leukemia. J Clin Oncol 42:3491-3503, 2024 39121442 10.1200/JCO.23.02238 PMC11458106

[41] Pölönen P, Di Giacomo D, Seffernick AE, et al: The genomic basis of childhood T-lineage acute lymphoblastic leukaemia. Nature 632:1082-1091, 2024 39143224 10.1038/s41586-024-07807-0 PMC11611067

[42] Li Z, Zhao H, Yang W, et al: Molecular and pharmacological heterogeneity of ETV6::RUNX1 acute lymphoblastic leukemia. Nat Commun 16:1153, 2025 39880832 10.1038/s41467-025-56229-7 PMC11779914

[43] Jeha S, Choi J, Roberts KG, et al: Clinical significance of novel subtypes of acute lymphoblastic leukemia in the context of minimal residual disease–directed therapy. Blood Cancer Discov 2:326-337, 2021 34250504 10.1158/2643-3230.BCD-20-0229 PMC8265990

[44] Mullighan CG, Goorha S, Radtke I, et al: Genome-wide analysis of genetic alterations in acute lymphoblastic leukaemia. Nature 446:758-764, 2007 17344859 10.1038/nature05690

[45] Fontana M, Zeni G, Vantini S: Conformal prediction: A unified review of theory and new challenges. Bernoulli 29:1-23, 2023

[46] Kumar B, Lu C, Gupta G, et al: Conformal Prediction with Large Language Models for Multi-Choice Question Answering, 2023. https://arxiv.org/abs/2305.18404

[47] Luo R, Zhao S, Kuck J, et al: Sample-Efficient Safety Assurances Using Conformal Prediction, 2021. https://arxiv.org/abs/2109.14082

[48] Yang K: Risk management in medical devices: An application of ISO 14971, in 2024 IEEE International Symposium on Product Compliance Engineering (ISPCE). Chicago, IL, IEEE, 2024, pp 1-3. https://ieeexplore.ieee.org/document/10541258/

[49] Wu E, Wu K, Daneshjou R, et al: How medical AI devices are evaluated: Limitations and recommendations from an analysis of FDA approvals. Nat Med 27:582-584, 2021 33820998 10.1038/s41591-021-01312-x

[50] Lysenkova Wiklander M: Molmed/allium_prepro: Initial release v1.1.0. 2024. https://zenodo.org/doi/10.5281/zenodo.14329215

[51] Lysenkova Wiklander M, Krali O: Molmed/allium: v2.4.0. 2024. https://zenodo.org/doi/10.5281/zenodo.14329233

[52] Lysenkova Wiklander M: Molmed/conformist: v1.1.1, 2024. https://zenodo.org/doi/10.5281/zenodo.14329248

[53] Lysenkova Wiklander M: Molmed/ALLCoP: v1.0.2, 2024. https://zenodo.org/doi/10.5281/zenodo.14333709

